# Sexual reproductive health service provision to young people in Kenya; health service providers’ experiences

**DOI:** 10.1186/1472-6963-13-476

**Published:** 2013-11-14

**Authors:** Pamela M Godia, Joyce M Olenja, Joyce A Lavussa, Deborah Quinney, Jan J Hofman, Nynke van den Broek

**Affiliations:** 1 Division of Reproductive Health, Ministry of Public Health and Sanitation, P. O. Box 30016, Nairobi, Kenya; 2 School of Public Health, University of Nairobi, P. O. Box 19676, Nairobi, Kenya; 3 World Health Organization-Kenya Country Office, P. O. Box 45335–00100, Nairobi, Kenya; 4 Research Methods, Liverpool School of Tropical Medicine, Pembroke Place, Liverpool L3 5QA, UK; 5 Sexual and Reproductive Health, Liverpool School of Tropical Medicine, Pembroke Place, Liverpool L3 5QA, UK; 6 Centre for Maternal and Newborn Health, Liverpool School of Tropical Medicine, Pembroke Place, Liverpool L3 5QA, UK

## Abstract

**Background:**

Addressing the sexual and reproductive health (SRH) needs of young people remains a challenge for most developing countries. This study explored the perceptions and experiences of Health Service Providers (HSP) in providing SRH services to young people in Kenya.

**Methods:**

Qualitative study conducted in eight health facilities; five from Nairobi and three rural district hospitals in Laikipia, Meru Central, and Kirinyaga. Nineteen in-depth interviews (IDI) and two focus group discussions (FGD) were conducted with HSPs. Interviews were tape recorded and transcribed. Data was coded and analysed using the thematic framework approach.

**Results:**

The majority of HSPs were aware of the youth friendly service (YFS) concept but not of the supporting national policies and guidelines. HSP felt they lacked competency in providing SRH services to young people especially regarding counselling and interpersonal communication. HSPs were conservative with regards to providing SRH services to young people particularly contraception. HSP reported being torn between personal feelings, cultural and religious values and beliefs and their wish to respect young people’s rights to accessing and obtaining SRH services.

**Conclusion:**

Supporting youth friendly policies and competency based training of HSP are two common approaches used to improve SRH services for adolescents. However, these may not be sufficient to change HSPs’ attitude to adolescents seeking help. There is need to address the cultural, religious and traditional value systems that prevent HSPs from providing good quality and comprehensive SRH services to young people. Training updates should include sessions that enable HSPs to evaluate how their personal and cultural values and beliefs influence practice.

## Background

The 1994 International Conference for Population and Development (ICPD) set the stage for putting adolescent sexual and reproductive health (SRH) on the international agenda. During the conference it was recognised that reproductive health needs of young people had largely been ignored by existing health, education and other social programmes. The conference adopted a plan of action which has formed the basis for programmes addressing the SRH needs of adolescents globally [1]. The five year progress review of this plan (ICPD + 5) made a further call for governments to ensure that adolescents have access to user-friendly services that effectively address their SRH needs including reproductive health information, education and counselling and health promotion activities, while encouraging their active participation [2]. A subsequent review conducted 10 years later (ICPD + 15) showed that teenage births were still a major concern, especially in sub-Saharan Africa where rates of more than 120 births per 1000 women aged 15–19 years are recorded and young people continue to be at risk for HIV infection, especially adolescent girls [3-5].

There is no single definition of SRH services but within the literature, SRH is described by the amalgamation of “sexual health” and “reproductive health”. Sexual health has been defined by the World Health Organization (WHO) as *“a state of complete physical, emotional, mental and social well-being in relation to sexuality; not merely the absence of disease, dysfunction or infirmity”*[6]. A positive and respectful approach to sexuality and sexual relationships is of paramount importance with the possibility of having safe sexual experiences which are free from discrimination, coercion and violence, and allowing for a sexual life that is safe and satisfying, with the freedom to decide if, when and how often to reproduce [1,6]. The ICPD Programme of Action included sexual health as part of the wider definition of reproductive health [1]. Adolescent SRH services therefore aim to provide information, education and health services to adolescents to help them understand their sexuality and protect them from unintended pregnancy and/or sexually transmitted infections including HIV/AIDS. It is recommended that this is combined with education of young men to respect women’s self-determination and to share responsibility with women in matters of sexuality and reproduction [1,7,8]. Youth friendly SRH services have been described by WHO (2002) as *“services that are accessible, acceptable, equitable and appropriate to meet the SRH needs of young people aged between 10–24 years.”* Such services are provided within an environment that is friendly and welcoming so that young people are able to come back again and also refer their friends for the same services [6]. Elements such as adolescent friendly policies, friendly health service providers and support staff, friendly service delivery mechanisms such as convenient opening hours, privacy and comprehensiveness of services have been cited as essential [6,7].

Kenya’s population is estimated at 38.6 million [8] and is projected to reach 56.5 million by the year 2025 [9]. Young people aged 15–24 years comprise almost 21 percent of the total population, out of which 51 and 49 percent are female and male respectively [8]. The Kenya Demographic Health Survey (KDHS) has shown a reduction in the percentage of teenagers aged 15–19 who have begun childbearing, from 23 percent (KDHS 2003) to 18 percent (KDHS 2008–09), with no difference between urban and rural populations.

Contraceptive use (any modern method) among sexually active girls aged 15–19 years, has increased from 20 percent in 2003 to almost 25 percent in 2008–09. Currently married women aged 15–19 mostly use the injectable contraceptive (14.4%), while unmarried women in the same age group commonly use the male condom (19.6%). Among currently married women, the unmet need for contraception among girls aged 15–19 years is 30 percent [9]. The HIV prevalence among young people aged 15–24 years is 3.8%, with women (5.6%) being four times more likely to be infected than young men of the same age (1.4%) [10]. Although the majority (90%) of young people aged 15–24 years know where to obtain an HIV test, less than five out of ten have ever gone for an HIV test and received a result [10].

Studies on sexually transmitted infections (STIs) among young people in Kenya are limited but current data shows that 12.6 percent of girls and 5.5 percent of boys aged 15–19 years are infected with HSV-2, while 0.6 percent of young people aged 14–24 years are infected with syphilis [10]. Young people are at increased risk of contracting STIs and in many countries age specific incidence and prevalence rates of STIs tend to be highest in the age group 15–24 years [11,12].

Girls aged 15–19 are twice as likely to die from pregnancy related complications compared to women in their 20s, while for girls aged 14 and below, this risk is increased fivefold [13]. In addition, children born of adolescent mothers are more likely to be underweight and die before their fifth birthday. Although adolescence is a stage in life where young people may be exposed to a number of risks and dangers, there is potential for promotion of healthy behaviour through appropriate education [14,15]. Behaviour initiated or learnt during adolescence may be long lasting and have either negative or positive influences on young people’s future lives. Efforts therefore have to be focused on promoting healthy and preventive behaviour during this stage of life [16].

Generally, HSPs approaches to addressing adolescent SRH have been found to be rather conservative in nature. Although nurses consider responding to adolescent sexual needs part of their routine nursing care, majority still face difficulties for example, in initiating discussions between adolescents while some feel discussions around sexuality should be the responsibility of the parents [17]. Adolescents are often not provided with the services they need, especially contraceptives and abortion, even where abortion is legal. Health providers have widely acknowledged the fact that they are not well equipped with knowledge and skills to effectively provide SRH services to adolescents [11,18].

A study carried out in Kenya and Zambia showed that nurse-midwives regard adolescent sexuality as a ‘moral issue’ and disapprove of adolescent pre-marital sex, abortion, and safer sex practices including contraceptive use [12].

A systematic review of interventions to increase young people’s use of health services in developing countries has shown that a combination of interventions, including health service provider training, facility improvement initiatives and community-wide health education can lead to increased service uptake. The need for careful monitoring, evaluation and operations research was also highlighted in this review [19]. Health care provider training on youth-friendly services (YFS) that are linked to other service components such as education in schools and the community, significantly increases service use especially among younger males (15-19 yrs) [20].

In the National Health Sector Strategic Plan II 2005–2010 (NHSSP II), adolescent SRH has been recognised as a priority within the Kenya Essential Package of Health (KEPH) [21]. Within the KEPH the Ministry of Health commits itself to providing services that are specific to this age group including reproductive health counselling, contraceptives and HIV/AIDS related services. This is to be achieved through the establishment of youth-friendly SRH health services within existing health facilities. According to the NHSSP II (2005–2010), the government intended to increase the number of facilities providing youth-friendly services from five in 2004 to 60 in the year 2010 [21]. In spite of this commitment, there is still some scepticism among planners, policy makers and development partners with regards to allocating resources to SRH services targeting young people. One of the reasons for this reluctance to allocate resources could be that stakeholders are not fully convinced about the model of service provision [13]. In addition to this, there is limited documentation on the state of SRH services for young people in Kenya.

This study was part of a larger research programme in Kenya, designed to explore the SRH needs of young people, perceptions of available SRH services from the perspective of young people themselves, community members and HSPs in order to provide more information on how best SRH services could be provided to young people in Kenya. This paper focuses on the perspectives and experiences of HSPs.

## Methods

SRH services for young people in Kenya are commonly provided through two service delivery models: youth centres and integrated health services. Youth centres only serve young people and can either be facility-based or community-based, while in the integrated health services model young people receive services within the general health system together with the general public.

Current estimates from the Kenya Service Provision Assessment Survey (KSPA) show that only seven percent of facilities are able to provide YFS, a decline from the 12 percent of facilities reported in KSPA 2004. However, 34 percent of the facilities have at least one health service provider trained in YFS provision [22].

### Selection of study sites and health facilities

The study health facilities were purposefully selected to include facilities where SRH services for young people had been in place for at least three years and ensuring the different types of model (health facility or youth centre) were included. Health facilities selected in Nairobi comprised of five facilities offering integrated services (four health centres and one clinic) and one youth centre. At the district level the sample included two district hospitals which had youth centres and for comparative purposes one district hospital that did not have youth-specific services, but where young people received SRH services as part of the general public (integrated services).

The study took place between the periods November 2008 – June 2009. In Nairobi, the five health facilities included three health centres, one clinic and one youth centre. At the district level the sample included two district hospitals (Laikipia and Meru Central), which have facility-based youth centres and for comparative purposes one district hospital (Kirinyaga) with integrated services.

Health service providers (HSPs) were selected using purposive sampling; a method mainly used in qualitative research that aims at selecting “information rich” respondents, who have extensive knowledge about a particular behaviour, experience or phenomenon of interest [23].

HSPs were selected taking into consideration: their firsthand experience in providing SRH services to young people and willingness to participate in the study. HSPs were selected from the following service delivery areas: MCH/FP clinics, youth centres, VCT clinics, maternity units, out-patient departments, comprehensive care centres (CCC), and obstetrics or gynaecology wards (Table 1).

**Table 1:** Details of study sites (n = 8) and selection of HSPs who participated in IDIs (n = 19) and FGDs (n = 18)

	**Number of health facilities or health service providers**	
	**IDIs**	**FGDs**
Total number of health facilities selected (n = 8)		
Nairobi	5	-
Districts	3	2
Facility type		
Integrated facilities	5	1
Youth centers	3	1
Health provider’s gender (IDI)		
Males	3	5
Females	16	13
Health provider’s cadre (IDI)		
Clinical officers	2	1
Nurse/Midwives	15	16
Counsellors	2	1
Health providers’ age (IDI)		
Average age	41 yrs	
Youngest	27 yrs	
Oldest	50 yrs	
Health providers’ years in providing SRH services (IDI)		Over 2 years
Average yrs of service	8.7 yrs	
Least yrs of service	2 yrs	
Most yrs of service	20 yrs	

Focus group discussions (FGDs) and semi-structured in-depth interviews (IDIs) were used as methods of data collection. The FGDs and IDIs with HSPs explored the following aspects of SRH service provision: knowledge of the available policies and guidelines, availability of SRH services, perceptions of and experiences with regards to SRH service provision, barriers to providing SRH services to young people and suggestions on SRH service improvement. The study tools were pre-tested in one of the health facilities in Nairobi that was not included in the study.

#### Ethics

Ethical approval was obtained from both the Liverpool School of Tropical Medicine Research Ethics Committee and the Kenyatta National Hospital Ethics and Research Committee. Before in-depth interviews were conducted, informed consent was obtained from each research participant, and a consent form signed as an indication of agreement to participate in the study. With regards to FGDs, verbal consent was obtained from all group members and only one consent form was signed by the FGD moderator to signify the group’s acceptance to participate in the study. This was done after an explanation had been given to the participants about the purpose of the study and the importance of their views as HSPs. Respondents were also given information sheets which had details about the purpose of the study. Permission was sought from all respondents to have the interviews and FGDs tape recorded.

#### Data analysis

A thematic framework approach was used to analyze the qualitative data collected [14]. The analytical process was systematic and followed the following five key steps [15]:

● Transcription of the tapes and field notes

● Checking and validating the transcripts

● Development of the thematic framework

● Coding of the transcripts using the thematic framework

● Charting and interpreting the data

While the five steps have been listed in a linear manner, the data analysis process was not always linear but rather it involved moving back and forth between the different steps in order to seek clarification of the responses and understand the context within which particular responses were made [14].

### Trustworthiness of the data

Trustworthiness is a term used in qualitative research to establish whether data collected during the research process is credible, transferable, dependable and confirmable [16]. Trustworthiness of the data collected in this study was met through triangulation of three aspects of data collection: i) having different respondents, ii) using different methods of data collection such as IDIs and FGDs, iii) using different researchers with different experiences to conduct interviews and moderate group discussions [24]. Member checking was also used for assessing the trustworthiness of the research findings [16].

## Results

A total of 19 IDIs and two FGDs with an additional 19 HSPs were conducted. Results are presented by the five key thematic areas that emerged: 1) HSP knowledge of policies and guidelines; 2) SRH services young people seek at health facilities; 3) HSPs’ views on SRH service provision; 4) barriers to SRH service provision; and 5) HSP suggestions regarding how SRH services can be improved.

### HSPs’ knowledge of polices and guidelines

HSPs from all study sites were aware of and had a relatively fair understanding of the YFS concept. HSP described YFS in terms of the way young people were approached, welcomed, listened to, handled, understood, given privacy and confidentially in order to facilitate free interaction.

*“These are services whereby a youth feels wanted, that is what I think it is or what I do to make the youth feel useful and there is a future for them and you make him feel good and he will be in a position to influence the other youths to seek services…*(16-IDI-HSP, Kirinyaga).

HSP from the districts identified some of the key features they thought were important if one had to provide SRH services to young people in a friendly way. This included understanding their language, the way of approach, having HSP who were younger, empathetic, knowledgeable and kind; but not motherly or fatherly.

Most HSPs were not aware of the national guidelines for provision of youth friendly services. A few HSP had seen or heard about them but were not sure of the content.

*“I have never* [seen the guideline]*, how long have they been there?…I have not seen any”* (01-IDI-HSP, Nairobi).

*“I am aware they are there but I cannot remember but I know there is a national guideline*”, (18-IDI-HSP, Laikipia).

The majority of HSP interviewed were aware of the RH rights young people have but there were some variations on how these rights were articulated. Most HSP reported that they were aware young people had a right to receive contraceptive services irrespective of age, they have a right to information on RH; for example if they want to use family planning they have a right to be counselled on both the advantages and disadvantages of each of the methods and thereafter make their own choice; they have a right to access other services such as STIs, Voluntary Counselling and Testing (VCT), and other HIV related services.

*“Well, they have a right to know their growth and development, they have a right to practice family planning if they feel they need it, they have the right to information, privacy and confidentiality, they also have the right to respect as young as they are, they need to be respected a lot…”* (15-IDI-HSP, Meru).

In contrast, during IDIs a few HSP from Nairobi reported that they were not aware of the reproductive health rights of adolescents or young people.

*“I have not heard about it really, I have not heard much about that, at least coz I know they have a right but I don’t know really about it in details”* (05-IDI-HSP, Nairobi).

### HSPs’ views on SRH service provision to young people

The majority of HSPs reported that young people visit health facilities when in need of the following services: contraception, condoms, abortion services due to unwanted pregnancy, treatment of complications due to incomplete or unsafe abortion, treatment of STIs, HIV services (VCT, ARVs) and treatment after sexual violence and rape. Other reasons mentioned were general counselling on social issues such as boy-girl relationships, misunderstandings between parents and young people and suicidal tendencies.

The perceptions and experiences of HSP in providing SRH services to young people can be categorized into three thematic areas: provision of contraceptive services; provision of STI and HIV related services and competency on SRH service provision.

### Provision of contraceptive services

The majority of HSPs were not comfortable providing contraceptives (excluding condoms) to young girls, especially Depo-provera, implants and IUCDs. Two reasons (beliefs) were given for this; i) contraceptives do not offer protection against STIs and HIV/AIDS; ii) contraceptives may adversely affect a young girls’ ability to conceive in future.

*“I would say according to my views I feel as a family planner they should not be given Depo [−Provera], it should be given to a person with known fertility…..things like IUCD, Norplant [are not acceptable]; youths should be restricted to pills only. Also, IUCD transmits STIs very fast………* (18-IDI-HSP, Laikipia)

Similar views were expressed by health providers in Nairobi where one HSP noted that:

*“Family planning should not be given to adolescents; they should be educated only because [family planning] is good for married people only.”* (10-IDI-HSP, Nairobi)

Some HSP reported being in a *“dilemma”* and indecisive on how to handle younger girls requesting contraception and reported that they discouraged these girls from taking up contraceptives.

*“….sometimes you wonder what to do for a 14 year old girl who needs contraceptives…”* (12-IDI-HSP, Nairobi).

HSP from both Nairobi and the districts often discouraged young people from using permanent contraceptive methods, irrespective of the number of children they had,

*“At the end of the day the choice is theirs, we give them the advantages and disadvantages to tell them that tubal ligation is a permanent method, it is irreversible and at 22 for them to decide, yes they might already have four kids, but there are long-term methods that they can maybe try, before they start talking about …tubal ligation”,* (05-IDI-HSP, Nairobi).

### Provision of HIV and related services

The majority of service providers, were supportive of the provision of HIV-related services to young people including VCT and ARVs. However, HSPs expressed concerns that young people were not adhering to the advice given to them during HIV counselling and testing. Young people were said to be coming for repeated HIV tests within an interval of a few days, giving reasons which HSP considered as flimsy such as *“niliteleza”* [I slipped, bad luck or unavoidable]. HSP reported being surprised when young boys (aged between 12–13 years) sought VCT services or were in need of condoms, HSPs speculated on whether such young boys were being sent by their older girlfriends.

*“Youth are very ignorant, first of all because even though they are given the information about anything, they are not serious about it, and especially the HIV area because…. you will find a youth coming for a test, especially HIV test, you counsel and then you see the same youth coming for the second and third time to be tested and then you wonder whether the youth is adhering to the information that you gave…… The major problem with them is the ignorance and they are not serious…”* (06-IDI-HSP, Nairobi).

Another HSP from Nairobi reported that some young people, after having consensual sex, came to the facility requesting for Post-Exposure Prophylaxis (PEP) on the pretext of having been raped.

*“…..they [young people] come, they say they have been raped, you try and ask questions to verify, not really that you are doubting, you are trying to verify the time, period, which ARV to use if they tell you how they were raped, for some of them at the end of the day you find that they had sex that they wanted to have only to realize later, they had made a mistake so they want post-exposure-prophylaxis but not because they were raped”,* (05-IDI-HSP, Nairobi).

HSPs from Nairobi also reported that they were receiving cases of teenage girls who turn out to be HIV positive despite reporting not being sexually active. Further in-depth investigation revealed evidence of peri-natal HIV transmission as their mothers turn out to be HIV positive upon investigation.

### Provision of condoms to young people

The majority of HSP give male condoms to young people either upon request by young people themselves or as part of the provision of advice on how to avoid STI, HIV and unwanted pregnancy prevention.

*“For the male [condom] if at all she ask me for it, I can give, and because she is asking for a family planning method. If at all, I test a client and she becomes [HIV] positive I normally provide the condoms so that they can use with the partner, .... so that I normally give them the condoms for prevention of STI and other problems.”* (04-IDI-HSP, Nairobi)

Female condoms were reported to be out of stock at all public health facilities for over two years. On the other hand, HSP also recalled the complaints made by clients on the use of female condoms, including discomfort, the noisy nature when in use and high cost.

*“Female condoms are out of stock, but most of the time I had heard them saying that it makes a lot of noise and it is not comfortable when you are wearing it.”* (03-IDI-HSP, Nairobi)

*“Even now as I was coming [for this interview] the last client I had wanted some female condoms, I showed her… and she said she has never seen them [before]. She was very impressed but it’s a bit expensive because I have told her the cost. They are more expensive than the male condoms and that could be the reason they are not available for us to give to clients.”* (06-IDI-HSP, Nairobi)

### Competency in SRH service provision

The majority of the HSP, especially from integrated facilities reported that they did not consider themselves competent in providing SRH services to young people. Most of them reported not having adequate counselling skills and not receiving any special training on how to handle adolescents.

*“I am not very competent because sometimes they come with so many issues and sometimes I am even defeated to answer some of the questions they [young people] ask me.”* (03-IDI-HSP, Nairobi)

*“I would like to say I am not really very competent, because sometimes you try to talk to them and sometimes the way they answer you, you feel like they are arrogant and they are like looking down upon you and really the communication, there is always a communication barrier, it’s very hard.” *(01-IDI-HSP, Nairobi)

*“….I have not done counselling so that could be a challenge to me, counselling is one of the main skills that I should have…..actually the techniques in making the youth to open up, at times they are quite protective, they can’t actually open up, so there is that barrier that the youth create which limits your access to them”* (01-IDI-HSP, Nairobi)

HSPs who had not received training in YFS indicated the need for more training especially in aspects of interpersonal communication, youth counselling, post-abortion care and post-rape care.

*“I would say I have the skills but I would need more because every time they [young people] come with something new, the youth have become very knowledgeable they would go to the internet and then they would come and challenge you with something you do not know or heard of” *(18-IDI-HSP, Laikipia)*.*

HSPs from integrated facilities in Nairobi who had not received training said that in serving adolescents, they used their skills as mothers, their experience from working in other departments and on-the-job-training they received from colleagues who had received training.

*“In particular, I have not been trained [in how to provide] youth-friendly [services] but from experience… I give what I have, though I don’t feel so competent, but I am trying as much as I can”* (02-IDI-HSP, Nairobi)

*“…but for me I have no training, being a mother I talk to them like being a mother”* (01-IDI-HSP, Nairobi)

*“…when you have people who have gone for training they come and give you feedback”* (04-IDI-HSP, Nairobi)

### Barriers to SRH service provision

**Figure 1:**
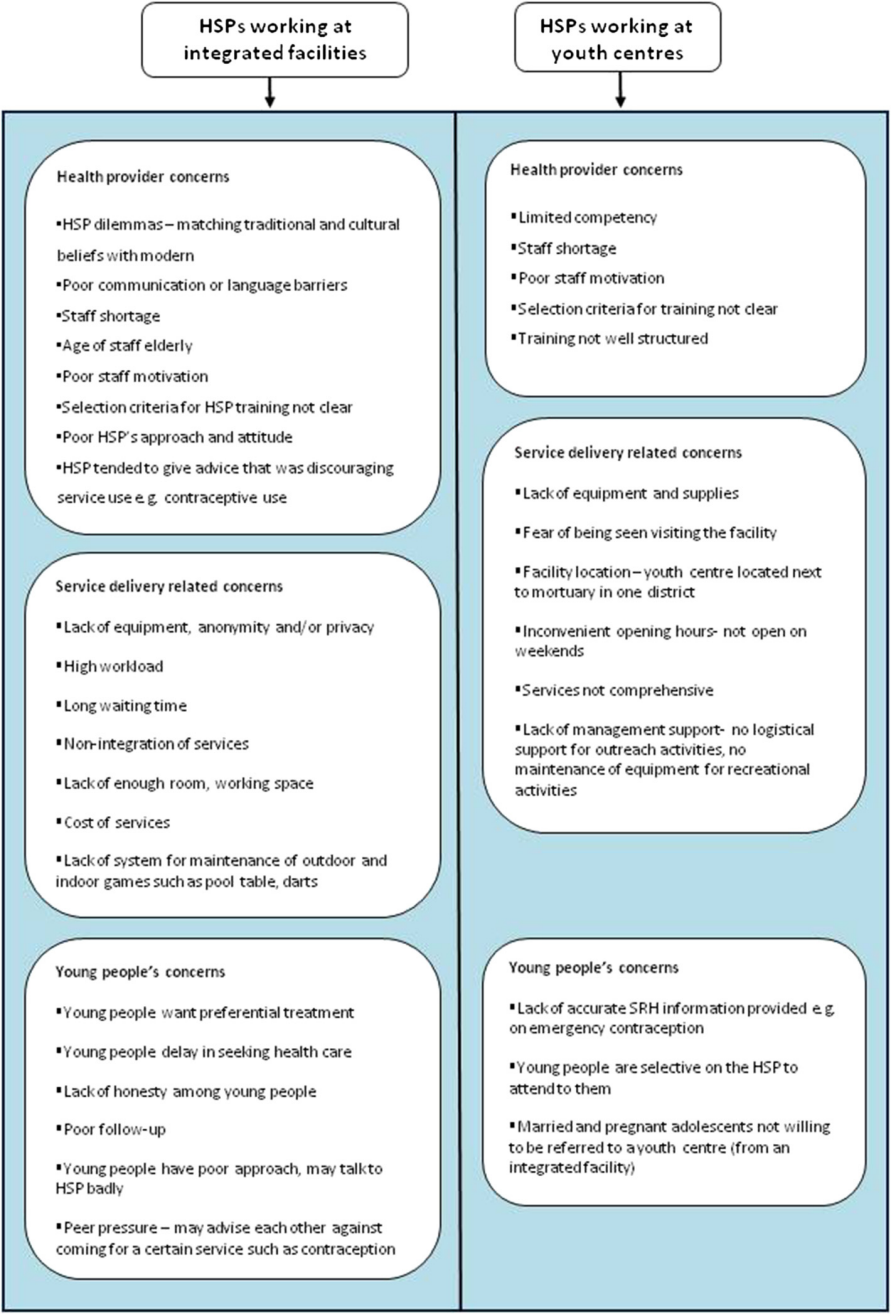
Health Service Providers’ (HSP) perspectives on barriers to sexual and reproductive health (SRH) service provision to young people.

HSP described their experiences as being either negative or positive. Negative experiences reflect barriers and challenges HSP experience while providing SRH services to young people. For both types of health facilities (integrated and youth centre), the barriers could be further classified into three broad thematic areas: factors that were related to the HSP themselves, the young people and the service delivery processes (Figure 1).

#### Health service provider related barriers

Limited knowledge and competency, HSP dilemmas, communication and language barriers, staff shortage, age of staff, poor staff motivation and selection criteria for HSP training barriers were commonly recognised and mentioned by the majority of HSP from all sites. The majority of HSP also expressed concerns about language and communication with young people, who often used *“ghetto”* and *“sheng”* languages which HSPs do not comprehend.

*“…Language also is another challenge like I had one who came and told me “manze kwa keja CD ikaburst”- I did not get it I heard [condom] burst only but the other [things]. I did not understand… so they are mostly used to talking sheng language which the [elderly HSP] do not understand”* (FGD-HSP, Laikipia)

HSPs from all facilities were concerned about the lack of clarity regarding the selection of HSP to attend training in youth-friendly services. HSPs from health centres in Nairobi indicated that some HSP who do not actually come into contact with young people (such as administrators) were given preference to attend training at the expense of HSP who actually serve young people.

*“…train the people who are on the ground so that they help the patients [rather] than training the bosses……every training, the [invitation] letter is taken to the in-charge [facility manager], the in-charge themselves attend the training and they are never there, so its misuse of the funds because it is not going to the client”* (01-IDI-HSP, Nairobi).

Also, HSP reported that colleagues were only interested in receiving training certificates “to keep in a box” as opposed to actually providing services.

*“If it is something [training in your area], you hear someone else has been picked from somewhere else and they have taken a course which is supposed to be [in your area], you feel de-motivated”* (FGD-HSP, Meru)

The majority of HSP, from integrated facilities, mentioned that a poor HSP approach and attitude tended to discourage service use and this included advice to adolescents not to seek SRH services. Examples of poor HSP attitude mentioned by HSPs themselves included being judgemental, having the tendency to easily condemn young people, being harsh and giving young people “lectures” when they presented with an SRH problem such as an STI or when in need of contraceptives or condoms.

*“It is true because I look at a young boy and girl who have come for condoms and they are the age of my son or daughter and I will ask, why are you taking them? And a young girl who has come for contraceptives then you start preaching to her or lecturing her and that is what they do not want.”* (11-IDI-HSP, Nairobi)

*“Or maybe you quarrel [with] them, where are you getting these diseases from? You are still young; do you know you can get HIV if you continue like this?”* (FGD-HSP, Laikipia)

*“Of course the age of the staff [is important]. When they meet somebody who is not of their age-group they say, I can’t tell this one she is just like my mother. So, of course, that way you don’t expect her to open up.”* (04-IDI-HSP, Nairobi)

**Figure 2:**
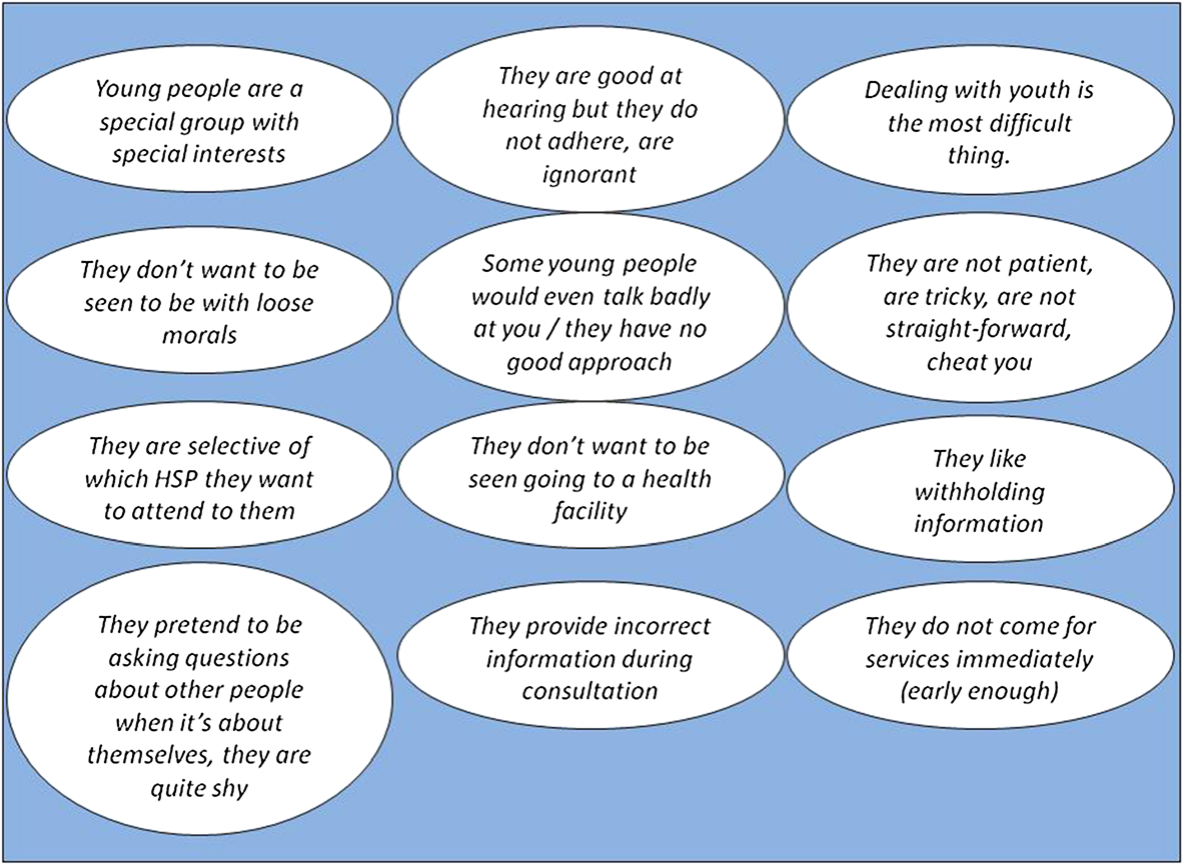
General descriptive terms used by health service providers (HSP) to describe young people.

Many HSPs reported that adolescents were not always easy clients and that the adolescents’ own attitude prevented them from receiving a good service (Figure 2).

#### Barriers to service delivery

Service delivery related barriers such as the lack of essential equipment and supplies (medication, contraceptives), lack of anonymity and privacy and high workload were barriers that were mentioned by the majority of HSP in all types of facilities. Long waiting times were mentioned by the majority of HSP from integrated facilities. Cost was a minority view that was elicited from integrated facilities in Nairobi, where one HSP said that although the cost of services had been subsidised, some young girls could not afford the minimum fee.

The majority of HSP from health centres in Nairobi reported the presence of long queues at the health facilities. In addition clients who needed in-depth discussion on particular issues were not well attended to as the contact time between HSP and young people was short.

*“…..time period, we have very long queues that is also another weakness, the youths are impatient, they don’t wait for long, some of them maybe have run away from home, they don’t want to be seen, all that contributes”* (05-IDI-HSP, Nairobi)

Inconvenient working hours, non-comprehensive services and lack of district management support were barriers mentioned by HSP from youth centres. At Nairobi youth centre, lack of full-time clinical staff meant that clinical services were only available three afternoons a week. The majority of HSP from all facilities were also concerned about the unavailability of a wide range of services; young people then had to be referred elsewhere.

### How to improve available SRH services

HSP were asked to make suggestions on how SRH services for young people could be improved (Table 2). HSP views have been categorised in three broad thematic areas to reflect whether the suggested improvements are targeting the HSPs themselves, the service delivery process or adolescents.

**Table 2:** Suggestions from Health Service Providers (HSP) on how to improve young people’s access to sexual and reproductive health (SRH) services

**Thematic area**	**HSP from integrated facilities**	**HSP from youth centres**
Health provider related improvements	▪ Improve staffing levels; appropriate staff deployment	▪ Improve staffing levels
	▪ Train more staff in youth friendly approach	▪ Train more staff in youth friendly approach
	▪ HSP training to include support staff	▪ Institute selection criteria for staff training
	▪ Selection criteria for staff training agreed	▪ Ensure staff motivated
	▪ Institute staff motivation and incentives such as certification, workshop attendance, recognition of good service	▪ Provide incentives for youth volunteers
	▪ Motivation of youth volunteers	▪ HSP to conduct outreach education activities in the community
Service delivery related improvements	▪ Ensure availability of essential supplies, drugs, basic equipment for Antenatal Care (ANC) and Family Planning (FP) Services; pregnancy tests kits, FP commodities, HIV testing kits	▪ Ensure availability of essential equipment in ANC and FP
	▪ Create adequate space to attend to young people	▪ Improve privacy of service provision
	▪ Need for facility improvement initiatives such as having youth-specific rooms where youth can be served without needing to queue	▪ Have full-time services i.e. 24/7 opening hours, or at least daily
	▪ Improve privacy in the consulting rooms	▪ Provide a wide range of services including ARVs, contraceptives, laboratory testing (e.g for STIs)
	▪ Have youth-only health facility/area if possible	
		▪ Set aside youth only days at the centre once a month
		▪ Introduction of pay-in service system for sustainability
		▪ Ensure that educational materials are available. Have these in audio-visual formats
Young people related improvements	▪ Need forums that can bring young people to the facility – youth-related initiatives and activities	▪ Hold youth-related activities at the facility
	▪ Have TV room with videos, library with education materials	▪ Have outreach activities to schools and churches
	▪ Have youth activities at the facility such as games, pool table, computers	▪ Have more activities at the centre to occupy young people

Improving staffing levels, staff training in YFS, instituting selection criteria for staff who attend training in YFS, and staff motivation were improvement areas that were mentioned by the majority of HSP from all the facilities. The need for forums or activities that bring young people to the health facilities was also mentioned. Ensuring availability of services, provision of integrated services, facility improvement initiatives and enhancing privacy and anonymity were mentioned by the majority of health service providers.

## Discussion

This study sought to explore health service providers’ (HSP) perceptions and experiences of providing sexual and reproductive health (SRH) services to young people in Kenya. Three main thematic areas emerged: HSPs’ understanding of youth friendly services (YFS), policies and guidelines, HSP training and provision of SRH services and HSP competency.

The findings in this study show some awareness of the YFS concept among HSP but not of supporting national policies and guidelines. This could either be as a result of unavailability of this information at the health facility level or lack of sharing of information by facility managers or HSPs who attend dissemination workshops. Dissemination of YFS provision policies and guidelines is therefore not very effective. These findings are in agreement with those reported in a study conducted in Swaziland, where 45 out of 56 HSPs reported lacking guidelines for YFS at their respective health facilities, while 9 HSPs reported having guidelines but not referring to them [11]. There is need for wider dissemination of available policies and guidelines to all levels of health care so as to ensure HSP are aware of, and understand, the guidelines informing service delivery. In addition, the content of these guidelines should be disseminated in a format that is easy to use by HSP such as via job aids, flip charts, CDs and DVDs. The process of developing service delivery guidelines should be all inclusive and allow participation of HSP themselves. This would enhance HSPs’ familiarity with the content and encourage ownership.

Irrespective of having received training in YFS provision, the majority of HSPs are reluctant to provide the full range of SRH services to young people. Although HSP know that in principle they should not deny young people any of the SRH services, their personal values and beliefs sometimes take precedence. This reluctance stems from HSP’s cultural, religious beliefs as well as perceived medical eligibility reasons. There seems to be a tension and contradiction between HSPs’ cultural and traditional values, existing policies and young people’s rights to receive SRH services. Although HSPs are supportive of policies that allow provision of SRH information and services to young people, at a personal level, they are often not comfortable actually providing services such as contraceptives to young people. Possibly HSPs feel and behave more as parents when dealing with adolescents and make their judgement from the perspective of their parental instincts and identity. Previous studies have also found that HSPs’ own cultural and religious values may hinder them from providing SRH services to young girls. A study in Swaziland [11] showed that culture, values and beliefs played a key role; for instance, emergency contraception was only provided to girls who had been raped, and only if they were brought in by the police [11]. A study carried out in Kenya and Zambia revealed that health workers who provide SRH services to adolescents face a dilemma between being true to their cultural and religious values and being sensitive to the needs of young people. Nurse-midwives regard adolescent sexuality as a ‘moral issue’ and disapprove of adolescent pre-marital sex, safe abortion and contraception use [12].

A systematic review of interventions to increase young people’s use of health services in developing countries has shown that multiple interventions, which include a combination of HSP training, facility improvement initiatives and community-wide health education, often lead to increased service use. This review also highlighted the need for careful monitoring, evaluation and operations research [19]. Evaluation of the National Adolescent Friendly Clinic Initiative (NAFCI) in South Africa found that agreeing on a set of adolescent friendly standards and criteria for service provision improved the quality of services, particularly, at clinics where a quality assurance facilitator was on-site and the intervention had been implemented for a longer period of time [25]. In Mozambique, training of health care providers from public health facilities together with improving facility infrastructure resulted in an increase in the number of young people accessing SRH services, especially girls who were coming for contraception and antenatal care services [26]. Evaluation of three youth-friendly projects in Zambia produced mixed findings, as increase in service use by youth could not be linked to the intervention at the youth centres, and was associated with “levels of community acceptance of the reproductive health services” [27]. Evaluation of youth centres in Togo showed an increase in youth centre use from 3.3% to 10.3% over three years. Contact with a peer educator, media exposure or close proximity to the youth centre was associated with increased uptake of services [28]. In Estonia, the setting up of youth clinics and training of health providers resulted in an increased number of girls visiting the clinics [29].

Overall, HSP training alone has been shown to result in only a modest increase in service utilization [20]. HSP training has also been found to be positively associated with improved management of other reproductive health problems such as provision of good quality post-abortion care in the event of an unsafe abortion [30]. Although HSPs have great reservations in providing contraceptives to young people, they are generally supportive of young people receiving other SRH services such as SRH education, HIV/AIDS related services, pregnancy-related services, treatment of unsafe abortion complications and treatment after sexual violence and rape.

Inappropriate selection of HSPs for training in YFS was emphasised by HSPs interviewed in this study. HSP are concerned that senior facility managers are often the ones selected (or rather they select themselves) to attend such training sessions while HSPs who deal with young people on a daily basis are left out. This has a direct effect on service provision as it de-motivates hands-on HSPs at the service delivery points. Similar concerns were raised in Tanzania where HSPs reported that the selection criteria for HSP training favoured senior managers who worked in administration and had minimal contact with young people [31]. There is therefore a need to institute good and fair selection criteria for training HSPs in YFS provision.

This study suggests that friendly policies and HSP training alone may not improve services. There is a need to address the cultural, religious and traditional value systems which prevent HSPs from providing comprehensive SRH services to young people. Training updates may not necessarily change personal attitude but can be used to enable HSPs to start evaluating their personal and cultural prejudices towards young people. There is a need to ensure that the training package is comprehensive and includes both theoretical and practical aspects of SRH service provision.

HSP training has been identified by WHO as one of the priority interventions for improving access to SRH services by young people [32]. The findings in this study show that HSPs often lack the knowledge and skills to provide good quality and comprehensive SRH services to young people. HSPs with no specific training use their skills as parents as well as their experience of working in clinical areas other than adolescent SRH.

HSPs’ difficulty in providing SRH services to young people could be presumed to originate from their inability to deal with young people at a personal level. This could reflect their poor understanding of adolescent psychology and recognised difficulty in providing counselling and interpersonal communication skills. This could also be reflective of deficiencies in the content of the existing SRH training curriculums. In Kenya the pre-service training curriculum for nurses has been revised to incorporate adolescent SRH concerns, but aspects of culture, the traditional belief and value systems may not have been adequately addressed. The Commonwealth Medical Association Trust (Commat) has developed a medical model curriculum on SRH to be integrated into undergraduate medical education and this could be adapted for use during continuing medical education sessions (CME) [33]. This curriculum highlights the need to include topics such as counselling and communication skills, disclosure of personal matters, health care provider attitude, and the influence of socio-economic and cultural factors on access to services [33]. Although the National Reproductive Health Training Plan for Kenya (2007–2012) advocates for on-the-job-training as an alternative way of improving the skills of HSP who have not undergone structured training as part of the pre-service curriculum, the effectiveness of this training will need to be assessed. There are currently no clear guidelines on how such training should be conducted to ensure competency and subsequent HSP certification [34].

We recognise the limitations of this study. Firstly, this is a qualitative study which took place in eight facilities and hence the results presented may not be generalised to the whole of the Kenyan population of health service providers. Secondly, the study has relied mostly on self-reported data. More research is needed to explore how socio-cultural and religious beliefs may act as either barriers or promoters of SRH service provision by health service providers.

The technical competencies of HSPs in providing YFS provision should be evaluated on a large scale, and improvements in the content of the curriculum for training HSPs undertaken to meet the gaps in knowledge and skills, especially with regards to adolescent psychology, counselling, and interpersonal communication. The training curriculum for HSPs should be revised to include aspects of culture, religion and traditional practices, with the aim of understanding their influence on HSPs’ decision making process, to provide SRH services to young people. Future research should focus on exploring how socio-cultural and religious beliefs hinder or promote SRH service provision by health service providers.

## Conclusion

Effective dissemination of existing policies and guidelines is essential for the harmonisation of service delivery at all levels of health care. Currently, HSP (even if trained) are still conservative with regards to providing SRH services to young people, especially contraception. Friendly policies and provider training alone may not improve availability of SRH services for young people. Cultural, religious and traditional values still play a major role in whether HSPs provide SRH services to young people. HSPs competency in providing SRH services to young people is limited by their understanding of adolescent psychology and their interpersonal communication skills. HSP seem to provide motherly, rather than professional, evidence-based advice and health services to young people.

## Competing interests

The authors declare that they have no competing interests.

## Authors’ contributions

GMP participated in the design of the study, development and pre-testing of the data collection instruments, training of the research assistants, conducting the interviews, checking and validation of the transcripts, data analysis, and drafting of this manuscript. OMJ participated in the training of the research assistants, validation of the transcripts, drafting and revising this manuscript. LAJ participated in data collection, and revising this manuscript. QD provided technical assistance on the methodology of the research, data analysis of revising this manuscript. HJJ provided supervision of the research project, participated in the study design, development of the data collection tools, analysis and interpretation of the results and drafting and revising of this manuscript. NvdB provided overall supervision of this research project, NvdB participated in the design of this study, development of the data collection tools, data analysis and interpretation of the results and drafting and revising of the manuscript. All authors read and approved the final manuscript.

## Pre-publication history

The pre-publication history for this paper can be accessed here:

http://www.biomedcentral.com/1472-6963/13/476/prepub
